# Why acute ischemic stroke patients in the United States use or do not use emergency medical services transport? Findings of an inpatient survey

**DOI:** 10.1186/s12913-019-4741-6

**Published:** 2019-12-03

**Authors:** Sudha Xirasagar, Meng-han Tsai, Khosrow Heidari, James W. Hardin, Yuqi Wu, Robert Wronski, Dana Hurley, Edward C. Jauch, Souvik Sen

**Affiliations:** 1 Department of Health Services Policy and Management, Arnold School of Public Health, University of South Carolina, Columbia, SC 29208 USA; 2 Department of Health, Human Services and Public Policy, California State University–Monterey Bay, Seaside, CA USA; 3 Blue Cross Blue Shield of South Carolina, Columbia, SC USA; 4 Arnold School of Public Health, Department of Epidemiology and Biostatistics, University of South Carolina, Columbia, SC, USA; 5 Bureau of Emergency Medical Services, South Carolina Department of Health and Environmental Control, Columbia, SC USA; 6 Genentech, Inc., South San Francisco, CA USA; 7 Department of Emergency Medicine, Department of Neurosciences, Mission Research Institute, Mission Health, Asheville, NC USA; 8 University of South Carolina School of Medicine and Prisma Health Midlands Richland Stroke Unit, Columbia, SC USA

**Keywords:** Emergency medical services transport, Ambulance use decisions, Acute ischemic stroke, Survey of stroke inpatients, Factors affecting patients’ EMS use decisions, Knowledge about stroke

## Abstract

**Background:**

Patients with acute ischemic stroke (AIS) who use emergency medical services (EMS) receive quicker reperfusion treatment which, in turn, mitigates post-stroke disability. However, nationally only 59% use EMS. We examined why AIS patients use or do not use EMS.

**Methods:**

During 2016–2018, a convenience sample of AIS patients admitted to a primary stroke center in South Carolina were surveyed during hospitalization if they were medically fit, available for survey when contacted, and consented to participate. The survey was programed into EpiInfo with skip patterns to minimize survey burden and self-administered on a touchscreen computer. Survey questions covered symptom characteristics, knowledge of stroke and EMS importance, subjective reactions, role of bystanders and financial factors. Descriptive and multiple regression analyses were performed.

**Results:**

Of 108 inpatients surveyed (out of 1179 AIS admissions), 49% were male, 44% African American, mean age 63.5 years, 59% mild strokes, 75 (69%) arrived by EMS, 33% were unaware of any stroke symptom prior to stroke, and 75% were unaware of the importance of EMS use for good outcome. Significant factors that influenced EMS use decisions (identified by regression analysis adjusting for stroke severity) were: prior familiarity with stroke (self or family/friend with stroke) adjusted odds ratio, 5.0 (95% confidence interval, 1.6, 15.1), perceiving symptoms as relevant for self and indicating possible stroke, 26.3 (7.6, 91.1), and bystander discouragement to call 911, 0.1 (0.01,0.7). Further, all 27 patients who knew the importance of EMS had used EMS. All patients whose physician office advised actions other than calling EMS at symptom onset, did not use EMS.

**Conclusion:**

Systematic stroke education of patients with stroke-relevant comorbidities and life-style risk factors, and public health educational programs may increase EMS use and mitigate post-stroke disability.

## Background

Chronic disease patients are at high risk for stroke (85% acute ischemic strokes, AIS), which affects over 800,000 Americans, causes about 142,000 deaths annually (5% of all deaths) and is the leading cause of chronic disability and long-term care costs[1–4]. Evidence is clear that AIS patients who receive quick reperfusion treatments with alteplase and endovascular thrombectomy have better survival and disability mitigation. Emergency medical services (EMS) transport is associated with greater likelihood of hospital arrival within the intravenous alteplase treatment window, 4.5 h since stroke onset[5]. Among patients arriving within the alteplase window, neuroimaging and reperfusion treatment are initiated more rapidly for EMS arrivals[6, 7]. These effects are mediated by various events triggered by EMS. EMS arrival itself attracts immediate attention of the hospital emergency medical team bypassing ED triage and wait times [8, 9]. Further, if advance notification of a brain attack (stroke, BAT) patient en-route is communicated by the EMS staff (BAT prenotification), the hospital stroke team is activated and convened ahead of the patient’s arrival [10, 11]. Prenotification also triggers clearing of the neuroimaging suite of non-emergent patients to receive the anticipated stroke patient immediately. Nationally, about 59% of AIS patients arrive by EMS[12]. However only about 30% of AIS patients arrive within the treatment window, with EMS patients being more likely than personal transport users to arrive within the treatment window[13]. Patients’ knowledge about stroke symptoms and the decision to promptly call EMS at symptom onset may be critical for survival and mitigation of long-term disability for some patients.

In the late 1990s, one-third of AIS patients were unaware of any stroke symptom, and one-third of AIS patients arrived within the 3-h clot-busting treatment window, and nearly two-thirds reported not being informed about their stroke risk before the episode. [14, 15]. Studies more than a decade later showed almost unchanged stroke knowledge and EMS use patterns despite sporadic mass media campaigns and despite mounting evidence of the life-changing impact of EMS use in professional scientific journals [16]. There is a need for empirical evidence on patient decision-making: what makes them use EMS or otherwise, in order to design appropriate interventions to maximize EMS use and mitigate post-stroke disability.

Qualitative studies show mixed findings on the factors that influence stroke patients’ decisions to use EMS. For example, a focus group of AIS survivors conducted after one year brought up financial concerns including potential claim denial by insurance as one factor in EMS use decisions [17]. A survey of AIS inpatients in the late nineties reported that neither insurance coverage nor out-of-pocket cost concerns influenced their actual EMS use decision [14]. Two studies including one from Australia reported that stroke symptom characteristics, family history of stroke, presence of a family member or bystander at the time of stroke, and cues received from a relative/bystander were associated with the decision to call for an ambulance [15, 18]. The absence of recent evidence on what makes United States patients use or not to use EMS has inhibited policies to improve EMS use by stroke patients. Valid and reliable data are best acquired from stroke patients surveyed as soon as possible following the stroke to minimize recall bias. However, surveying patients during their stroke hospitalization is challenging, given their acute illness and disability status, unstable medical condition and preoccupation with intensive care procedures. This study presents the findings of a convenience sample survey of AIS patients who were surveyed during hospitalization to capture the facilitators and barriers that led to their EMS use decisions.

## Methods

We conducted a convenience sample survey of hospitalized patients admitted with a primary diagnosis of AIS to a primary stroke center of a nonprofit, medical school-affiliated hospital in South Carolina. The survey was conducted concurrent with a prospective observational study of the impact of EMS use on patient disability outcomes.

### Survey development, pretesting and administration

A survey was developed based on a draft conceptual framework of the potential decision drivers of EMS decisions developed from the documented literature (which is sparse) and expert input gathered by brainstorming with stroke care providers [14–18]. the neurologist (study coinvestigator and manuscript coauthor), hospital stroke unit nurse coordinator, stroke unit floor nurse, Stroke Rehabilitation and Physical Therapy unit Director (a physical therapist by training), and the hospital’s GWTG-Stroke registry staff who track patients through the various unit/floor transfers until discharge using an intranet-enabled patient tracking tool. To incorporate perspectives of field EMS staff and to adapt the survey wording to the general population reading level, the resulting draft survey was reviewed jointly with the Chief of the South Carolina Emergency Medical Services Bureau, a trained EMS technician with field EMT experience who supervises EMS operations in South Carolina (also a co-author of this study). The final draft survey was adapted for 5th grade reading level, presented in Additional file 1. The Patient Consent form was adapted from the study hospital’s IRB-cleared consent template that is used for consenting patients for clinical trials and interventional studies.

After finalization, the PI trained the pilot survey administrator (a stroke unit nurse experienced in consenting patients for clinical trials). The PI and the stroke nurse reviewed the survey jointly item by item and developed consensus on responding to potential patient questions and concerns. Following training, the survey (programmed into a touchscreen computer) was pretested by the stroke nurse with 10 patients to resolve ambiguities in question wording, language above patient comprehension level, and question order glitches. Following pretesting, a graduate student assistant and stroke registry staff were trained (the latter performing survey work during after-hours to fill in during the university summer and winter breaks). Because survey format issues during pretesting were minor not germane to data integrity, the pretest surveys (10 surveys) were included in the final data for analysis.

The survey was programmed into EpiInfo (a software package offered by the Centers for Diseases Control and Prevention) in a touchscreen Microsoft Surface tablet. Questions were set to progress to the next applicable question. In almost all cases, patients completed the survey on their own, occasionally seeking clarification from the survey staff who stood by. We chose computer-based self-administration to enable patients to freely respond to sensitive questions (e.g. alcohol, drug use, medical mistrust). Graduate student assistants (2 graduate students over the course of the study) who administered the survey were additionally trained in determining patient eligibility for survey from the online (intranet) Patient Tracking tool updated each day by the Registry staff, and to contact the floor nursing staff to obtain clinical fitness clearance to interview the patient.

### Survey content overview

**Fig. 1:**
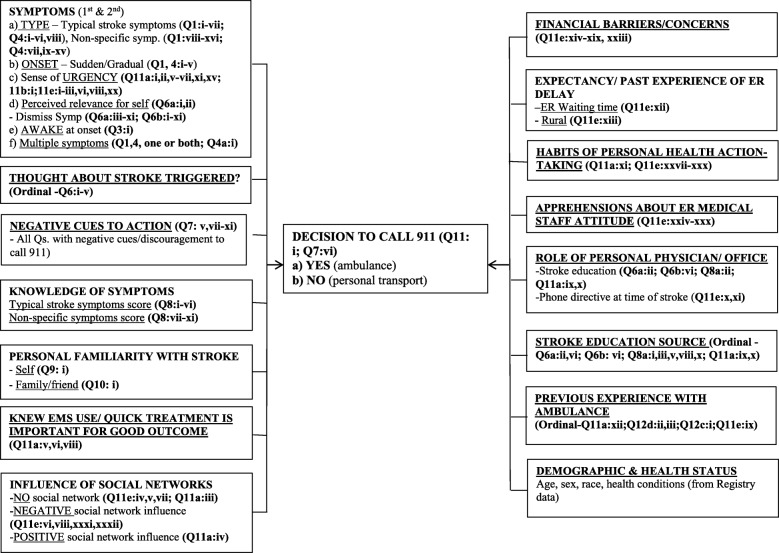
Potential clinical, subjective and contextual factors influencing stroke patients’ decisions to call 911 for an ambulance*. *Refer to Additional file 1, Patient Survey instrument to identify the questions marked by Question numbers in parentheses in the figure. Sum of item responses (yes/no) used to compute each factor

The final survey covered the stroke symptoms that were experienced, prior knowledge of symptoms, prior familiarity with stroke (personal history or family/friend with stroke), bystanders’ role in the decision to call EMS, financial barriers to EMS use, role of their personal physician/office staff in stroke education, role of cues given by the physician’s office when patients called the office at stroke onset, patients’ current and prior experiences with EMS and hospital emergency departments, patients’ subjective apprehensions/expectations of provider reactions and medical institutional trust, and personal health habits. Figure 1 shows the conceptual framework of potential facilitators and barriers that may influence 911 call decisions. The survey is presented in Additional file 1.

Symptom-related questions included: type and multiplicity, severity, onset (sudden or gradual), and awake (or otherwise) at stroke onset. Prior stroke knowledge questions included: knowledge of typical and atypical stroke symptoms, match between known symptoms and experienced symptoms, knowledge about the importance of EMS transportation for quick treatment and good outcome, source of stroke knowledge, and perceiving symptoms as relevant for self or being dismissive of symptoms. Patients were asked to select and rank up to five priority reasons for calling or not calling 911, and to provide recommendations to improve EMS use by the stroke population. A pre-developed list with an open-ended option was presented.

Responses were captured into a background Microsoft Excel database. Survey items were grouped to represent a purported facilitator or barrier to EMS use as shown in Fig. 1 and summary scores calculated for each construct by adding item scores (yes = 1, no or not selected = 0).

### Patient sample and data collection

We surveyed a convenience sample of patients admitted between July 5, 2016 through March 12, 2018. Eligible patients were those with documented neuroimaging evidence of AIS (identified from the hospital’s Get-With-The-Guidelines (GWTG) stroke registry system), patient not assigned to hospice care at admission, not in ICU when contacted, physically and clinically fit to participate (cleared with floor nurse), and not engaged with medical procedures or social/family visits when contacted (during business hours, Monday to Friday). The hospital Stroke Unit’s Patient Tracking tool, routinely used by GWTG-registry staff to monitor patients’ intrahospital status and transfers, was supplemented with additional data fields for study purposes and uploaded daily to the hospital intranet to facilitate identification of survey-eligible patients. During business hours, survey staff contacted survey-eligible patients (i.e. National Institutes of Health Stroke Scale [NHISS] score of < 15 at contact and not in the intensive care unit). Survey eligible patients included patients with speech difficulties but otherwise mentally fit to participate with a relative’s assistance. Multiple attempts per patient were made until discharge.

Written informed consent was obtained and respondents were provided a $20 departmental store gift card upon survey completion. The study was approved by the hospital Institutional Review Board (IRB). Each survey was assigned the patient’s link identifier, a random identification number assigned to all AIS patients of the study period by GWTG registry staff. The identifier enabled survey linkage with demographic and clinical data.

### Data analysis

Descriptive statistics were used to study patient characteristics and the self-reported prevalence of facilitators and barriers. Two-variable logistic regression analyses were performed to assess the severity-adjusted (admission NIHSS score) association of EMS use with the facilitators and barriers. Multivariable logistic regression analysis was performed to further clarify the adjusted significance of the barriers and facilitators (stepwise manual variable selection). *P* < 0.05 was considered statistically significant. Analyses were performed using SAS version 9.4 (SAS, Cary, NC).

## Results

Of 1179 admitted AIS patients, 108 were medically and physically fit, available when contacted, and consented to complete the survey, including 24 who completed it with a relative’s assistance. Of survey respondents, 11 were transfer patients from another hospital and lacked admission stroke severity and demographic data (extracted from the GWTG registry database). Surveyed patients were slightly more likely than those not surveyed to be female, younger, White/other race, slightly less likely to have severe stroke, and somewhat more likely to have received alteplase reperfusion treatment. (Additional file 1).

Table 1 presents respondent characteristics and responses to key questions. Among total respondents, 49% were male, 44% African American, 59% had mild stroke (admission NIHSS ≤5), median admission NIHSS score was 4.0, mean age 63.5 years, 33% unaware of any stroke symptom before the episode (similar among EMS users and non-users), and 75% were unaware of the importance of EMS use for better outcomes. EMS users differed from non-users as follows: all patients who knew the importance of EMS had used EMS. None of the non-users knew the importance of EMS use. Significantly more patients who had prior familiarity with stroke (through a personal or family member/friend’s stroke experience) had used EMS (80% vs. 58%), as did patients who were awake at stroke onset (80% vs. 64% of wake-up strokes), those who were encouraged by bystanders to use EMS (family/friend/neighbor) (44% vs. 0%), and those with prior experience of EMS use (67% vs. 36%). More EMS non-users than users reported financial/cost concerns (30% vs. 15%). Eight out of 10 patients who were discouraged from using EMS by bystanders did not use EMS. All 6 patients who called their physician’s office at symptom onset and were directed to come to the physician office or to go directly to the emergency department (ED) did not use EMS. Of EMS non-users, 27% reported living out in the country and perceived personal transport to be a quicker option, and 6% reported using personal transport because they anticipated care delays in the ED anyway. Responses to the complete list of survey questions on the facilitators and barriers are presented in Additional file 1.

**Table 1:** Characteristics and Responses of Surveyed AIS Patients

	All surveyed patients (*n* = 108) No (%)	Classified by EMS use	
		Yes (*n* = 75) No (%)	No (*n* = 33) No (%)
Patient characteristics			
Sex^a^*			
-Male	47 (48.5)	31 (45.6)	16 (55.2)
-Female	50 (51.5)	37 (54.4)	13 (44.8)
Age, mean (SD)	63.5 ± 15.4	64.3 ± 15.4	61.6 ± 15.6
Race^a^*			
-White/Asian/Other	54 (55.7)	32 (47.1)	22 (75.9)
-Black/African American	43 (44.3)	36 (52.9)	7 (24.1)
Severity based on initial NIHSS^a^*			
-Mild (NIHSS 0–5)	57 (59.4)	33 (49.3)	24 (82.8)
-Moderate (NIHSS 6–15)	27 (28.1)	23 (34.3)	4 (13.8)
-Severe (NIHSS ≥16)	12 (12.5)	11 (16.4)	1 (3.4)
Alteplase at study hospital^a^*	14 (14.4)	14 (20.6)	0
Comorbidities/Risk factors^b^, mean (SD)	2.4 ± 1.5	2.4 ± 1.4	2.4 ± 1.7
Response to selected survey questions			
Symptoms			
-Had ≥1 typical stroke symptom	90 (83.4)	62 (82.7)	28 (84.9)
-Thought of stroke and perceived symptom as relevant and indicating possible stroke (not dismissing the symptom)*	74 (68.5)	67 (89.3)	7 (21.2)
-Awake at stroke onset	81 (75.0)	60 (80.0)	21 (63.6)
Knowledge of symptoms			
-Knew some typical stroke symptoms	72 (66.7)	48 (64.0)	24 (72.8)
-Knew no symptom	36 (33.3)	27 (36.0)	9 (27.3)
-Familiar with stroke experience due to personal history or family/friend with stroke*	79 (73.2)	60 (80.0)	19 (57.6)
Knew the importance of quick treatment /ambulance arrival for good outcome*	27 (25.0)	27 (36.0)	0
Influence of social networks			
-Family member/bystander discouraged patient from calling 911*	10 (9.3)	2 (2.7)	8 (24.2)
-Family member/bystander supported patient thoughts to call 911*	33 (30.6)	33 (44.0)	0
Reported financial concerns about ambulance use/concern about cost of ambulance use	21 (19.4)	11 (14.7)	10 (30.3)
Prior experience of or expectation of long ER wait time*	2 (1.9)	0	2 (6.1)
Live out in the country, better to drive personally to reach quickly*	10 (9.3)	1 (1.3)	9 (27.3)
Role of personal physician or their staff			
-Patient reported being educated about stroke symptoms by their doctor or nurse	37 (34.3)	27 (36.0)	10 (30.3)
-Physician’s office directed the patient to actions other than calling 911 when symptoms occurred*	6 (5.6)	0	6 (18.2)
Source of stroke knowledge			
-Physician/nurse/personal stroke experience	55 (50.9)	36 (48.0)	19 (57.6)
-Public sources (internet, billboards, etc.)	30 (27.8)	23 (30.7)	7 (21.2)
-No stroke knowledge	23 (21.3)	16 (21.3)	7 (21.2)
Previous experience with ambulance			
-Had prior experience of self/family members with calling 911 for ambulance*	63 (58.3)	51 (68.0)	12 (36.4)
-Had a bad ambulance use experience	3 (2.8)	2 (2.7)	1 (3.0)
Concerns about ED medical staff’s negative affective response due to personal health habits or other reasons	0	0	0

AIS, acute ischemic stroke; EMS, Emergency Medical Services; ER, emergency room; NIHSS, National Institutes of Health Stroke Scale

^a^ Patients with missing sex, age, race, and NIHSS data were transfer patients from another hospital

* *P* < 0.05 between EMS and non-EMS groups

^b^GWTG (Get With The Guidelines)stroke-relevant conditions/risk factors: atrial fibrillation/flutter, coronary artery disease/prior myocardial infarction, carotid stenosis, depression, diabetes mellitus, drugs/alcohol abuse, dyslipidemia, heart failure, hypertension, migraine, obesity/overweight, previous stroke, previous transient ischemic attack, peripheral vascular disease, renal insufficiency, sleep apnea and smoking history

Responses to the complete list of survey questions are presented in Additional file 1

Patients reported their priority reasons for using or not using EMS, shown in descending order of frequency in Table 2 (multiple reasons per patient, total adds up to more than 108). Severe, sudden or scary symptoms were reported as a key reason they used EMS by 63% of EMS users, and conversely, 21% of non-users reported mild, gradual or fleeting symptoms as a reason for not using EMS. Among EMS users, knowing the importance of EMS for a good outcome was reported by 26% as a key reason, as was their doctor’s prior advice to call 911 if stroke symptoms occurred (8%). For 31% of all surveyed patients, the bystander’s suggestion/insistence was a key reason for EMS use. For 5% of all patients, bystander discouragement to call 911 was a key reason for not using EMS. Other key reasons why non-users did not use EMS included cost or insurance concerns, contrary advice by their doctor’s office (either to go to the hospital directly or come to the doctor’s office), and, living in the countryside causing them to believe personal transport as the quicker option (7% each).

**Table 2:** Patient-reported Facilitators and Barriers for EMS Vehicle Use (selections out of an itemized list; multiple reasons per patient, total adds to more than 108)

Patient-reported priority reasons for using or not using EMS	No (% of total 108 surveyed patients)
Facilitators/reasons why patient used EMS^a^ (75 patients who used EMS)	
A neighbor or family member agreed/insisted to call 911	33 (30.6)
Symptoms were severe and scary	31 (28.7)
I felt my symptoms could be stroke	31 (28.7)
I knew that arriving at the hospital quickly was important	16 (14.8)
I felt ambulance was the best way to get care	9 (8.3)
My doctor/nurse told me to call 911 if I had symptoms	9 (8.3)
I was unconscious; a bystander called 911	6 (5.6)
I know someone with bad effects of stroke due to not calling ambulance	2 (1.9)
I normally take care of my health and felt I needed care urgently	2 (1.9)
I knew others who became disabled or died from stroke	1 (0.9)
I/We had good prior experience with using ambulance	1 (0.9)
Barriers/reasons why EMS was not used^a^ (33 patients who did not use EMS)	
Symptoms took a long time to become serious	10 (9.3)
I live out in the country, driving may be quicker than ambulance	8 (7.4)
I felt normal; symptoms came and went	7 (6.5)
I had no pain, so I did not feel it was urgent or serious	6 (5.6)
I called my doctor and they asked me to go directly to the hospital ER	6 (5.6)
Family member present insisted we should not call ambulance	5 (4.6)
I was at work/somewhere else and waited till I could leave	4 (3.7)
I did not know that I could have serious problems if not treated quickly	2 (1.9)
I have no insurance, will get a big bill/worried about my share of ambulance cost/insurance may not cover if symptom is not serious	5 (4.6)
I/my family member had a large medical expense or bills before this	2 (1.9)
There is a long waiting time at the ER anyway, so might as well go by car	2 (1.9)
The ambulance siren and lights will disturb neighbors, and I don’t want them to know my business	1 (0.9)
My family members were out	1 (0.9)
I live alone and was too weak to call	1 (0.9)

*EMS*, emergency medical services, *ER* emergency room

^a^ Aggregated from the top three self-reported facilitators for ambulance use among those who used ambulance and top three self-reported barriers for ambulance use among those who did not use ambulance. Up to 3 priority reasons included in the table. Numbers add up to more than 108

Regression analysis showed that admission stroke severity was the outstanding driver of EMS use. After adjusting for stroke severity (admission NIHSS used as a continuous variable) the results of 2-variable regressions assessing each factor are shown in Table 3. Patients who perceived the symptom as relevant and indicating possible stroke were more likely to have used EMS (adjusted odds ratio [AOR] 26.3 (95% CI 7.6, 91.1), as were patients who were awake at stroke onset (AOR 3.6, [1.2, 11.0]), and those with prior stroke familiarity (AOR 5.0, [1.6, 15.1]). Patients who were discouraged from calling 911 by bystanders were 90% less likely to call EMS (AOR 0.1, [0.01, 0.7]). Most factors retained statistical significance in multiple regression analysis (Additional file 1).

**Table 3:** Two-variable Regressions Showing the Factors Associated with EMS Use after Adjusting for Stroke Severity^†^

	Adjusted Odds Ratio (95%CI)
Stroke symptom characteristics	
Awake at stroke onset: Yes (vs. Not awake at onset)*	3.6 (1.2, 11.0)
Multiple symptoms at onset (vs. Single symptom at onset)	6.1 (0.1, 363.0)
Knowledge/familiarity with stroke	
Familiarity with the stroke experience (Self or family member/friend had a stroke): Yes (vs. No)*	5.0 (1.6, 15.1)
Thought of stroke and perceived symptom as relevant for self: Yes (vs. No)*	26.3 (7.6, 91.1)
Perceived external barriers to EMS use	
Family member/other person present discouraged calling 911: Yes (vs. No)*	0.1 (0.01, 0.7)
Reported financial barrier/concern about cost of ambulance use: Yes (vs. No)	0.4 (0.1, 1.2)

EMS, emergency medical services; OR, odds ratio

† Some factors did not show statistical significance due to zero or very low values in one of the EMS use categories. These were: Knew the importance of quick treatment/ambulance arrival, Family member/others around at time of stroke, Positive encouragement to call 911 by bystander, Previous experience or expectation of long ED wait anyway, Live out in the country, Tend to be proactive about personal health, Concerned about ED medical staff’s negative affective response, Directed to actions other than calling 911 by physician’s office, and Prior experience of self/family members with 911 for ambulance

* *P* < 0.05 for decision to call 911 for an ambulance (Yes/No), adjusted for stroke severity, admission NIHSS score

Current and past EMS experiences are presented in Additional file 1. Of total respondents, 63 patients (58%) had used EMS previously; a third had paid for most or all the EMS cost out of pocket. A negligible number of patients reported negative or discriminatory experiences with EMS staff or ED physicians. Notably, six patients refused the ambulance called by a bystander.

Patient recommendations for increasing EMS use were: full insurance coverage without out-of-pocket cost (46%), stroke education of high-risk patients by doctors (43%), educational brochures at doctor’s offices (39%), and television commercials (39%) (Additional file 1).

## Discussion

The study uncovered key findings for public health and provider-driven interventions to improve EMS use by acute stroke patients. The validity of findings is enhanced by concurrence of patient-reported hospital arrival mode and hospital record-documented arrival mode, as well as survey administration soon after admission, minimizing recall bias (interquartile range of admission to survey interval, 2–5 days). One study limitation is the proportion of AIS inpatients who were surveyed, 9.8%. Two structural barriers interacted to impede survey coverage: a) limited availability of survey staff time (graduate research assistants available for 20 h a week, and patchy survey staff coverage during university breaks (stroke unit/stroke registry staff working after-hours), and, b) limited patient availability primarily due to short inpatient stay (as a rule, patients are discharged to skilled nursing facilities after acute care needs are met). AIS patients’ median length of stay during the study period was 5 days (45% were discharged on the 4th day or earlier). Stroke patients typically experience intensive medical care in the first 2–3 days (unavailable for survey) or they are medically unfit, being in the acute recovery phase. Consistent with these conditions, survey staff found 34% of patients were already discharged at their first attempt to contact the patient. Other reasons for not contacting the patient were: not contacted (weekends, university breaks), patient asleep, transferred to ICU, expired or assigned to hospice care. The distribution of survey-eligible patients by survey status, and reasons for non-completion are presented in Additional file 1. Given the transient availability of this acute care population and reasons for non-completion, far more intensive resource expenditures are needed to achieve a significantly better survey completion rate.

The observed minimal differences between surveyed and not-surveyed patients on most demographic and stroke characteristics, and similarity of surveyed patients’ stroke severity to that of recent nationwide AIS cohorts (median admission NIHSS score nation-wide, 4.0), suggest that the evidence produced by this study can be used for public health and medical initiatives to increase EMS use by stroke-symptomatic patients [13]. The validity of findings is further supported by internal consistency of survey responses across survey sections. For example, patient-reported priority reasons for their EMS decisions were validated by predictive modelling using their responses from other survey sections, and patients’ reported priority reasons for use or non-use of EMS were correlated with their recommendations to improve EMS use by stroke patients.

As anticipated, symptom severity was the most critical factor in EMS use. Regression analysis showed 20% higher odds of EMS use per unit increase in the NIHSS score. EMS use was also highly driven by patien perception of the symptom as relevant for self and as a possible stroke symptom (i.e., not being dismissive of the symptom), prior familiarity with stroke, and prior knowledge of the importance of EMS use and quick treatment for a good outcome. Factors that reduced the likelihood of EMS use were mild, fleeting or vague symptoms and the absence of pain (total of 21% of patients, Table 2), bystander discouragement to call 911, confusing or contrary directives from their physician office, and living out in the countryside causing them to go directly to the hospital to save time. Most patients (83%) had multiple typical stroke symptoms at onset, yet only 69% used EMS. These findings indicate a serious knowledge gap about stroke among patients who are at higher risk of stroke due to 15 risk factors (atrial fibrillation/flutter, coronary artery disease/prior myocardial infarction, carotid stenosis, depression, diabetes mellitus, dyslipidemia, heart failure, hypertension, migraine, obesity/overweight, previous stroke, previous transient ischemic attack, peripheral vascular disease, renal insufficiency, sleep apnea, smoking, and drug/alcohol abuse,). Findings on bystanders’ role in the decision reveals stroke knowledge gaps among the general population. These findings call for systematic engagement of providers in stroke education of high-risk patients, and systematic public health education programs with population-wide outreach.

Our finding that a third of patients were unaware of any stroke symptom before the episode, and nearly three-fourths were unaware of the importance of EMS suggest a lack of progress on stroke education since 1997 and 2010, when studies showed similar levels of ignorance about stroke and action-taking when faced with stroke symptoms [14–18]. In both prior studies, one-third of patients reported being educated by their doctor about stroke symptoms and stroke risk. These findings are also similar to a 2005 study [16]. However currently, a major messaging challenge for education programs must be noted. The overwhelming factor driving EMS use decisions was symptom severity; however, over 50% of strokes (both in this study and nationally) are mild strokes (i.e. admission NIHSS 0–5, although some mild stroke could deteriorate if not treated promptly). Both nationally and in our surveyed sample, the median NIHSS score was 4.0. For both physicians and public education programs, a key communication challenge is how to effectively communicate to the population about the critical importance of EMS use despite mild symptoms.

Patients’ subjective determination that their symptom was relevant for themselves as a possible stroke symptom (i.e. not dismissing the symptom) was another key factor driving EMS use. This finding is similar to a 1997 study in North Carolina with a similar inpatient sample and adjustment for admission stroke severity [14]. On a cautionary note, however, our findings may reflect unresolved messaging challenges rather than inadequate educational efforts by providers -- how to successfully convey complex, future-stroke risk and EMS information in a typical 15 to 20-min office visit of a multi-comorbid patient. Patients’ ignorance may also reflect how they prioritize their listening and information processing which may focus on pressing medical symptoms to the detriment of information on future disease risks. Regardless, the persisting knowledge gaps reinforce the need for major public health and medical/nursing community efforts to develop effective communications and outreach strategies to educate co-morbid patients and those with lifestyle risk factors, as well as the general population. Innovative communication strategies including technology-assisted approaches may help address this challenge. Another approach may be for professional medical and nursing organizations to include stroke education as a standard of care in chronic disease and lifestyle risk factor management guidelines.

Three barriers to EMS use that failed to attain statistical significance in adjusted analyses (due to zero frequency among EMS users) are nevertheless important: patients receiving confusing or contrary directives from their physician office (none used EMS); patients unaware of the importance of EMS use (none used EMS), and peri-urban residents perceiving personal transport as the quickest option (10 out of 108 patients, none used EMS). Practice implications of these findings are as follows. There is a need to train physician office staff in recognizing emergent symptoms communicated by phone, and to clearly direct patients to call EMS. The finding regarding peri-urban residents needs further research. Because we did not extract residential zip code data, the magnitude of this factor among total rural/peri-urban respondents is not known. The messaging implication of this finding remains unclear, because there is no documentation whether EMS use is helpful or harmful due to time considerations among this sub-population.

Interestingly, although a fifth of respondents considered the financial burden of EMS costs before making their decision, it was not reported as a priority reason for their decision. Regression analyses also showed that it was not a barrier for EMS use, similar to a North Carolina study [14]. However, almost 50% of surveyed patients, recommended insurance mandates for full coverage of EMS when used by stroke-symptomatic patients. Their recommendation may reflect prior experience with EMS use: 58% of surveyed patients had used EMS in the past, with one-third of them incurring most or all EMS costs out-of-pocket. Other recommendations by respondents were: patients should be educated by their doctors on stroke and the importance of EMS, educational brochures should be placed in doctors’ offices, and television commercials broadcast.

The study had some limitations. Survey completion may be mentally challenging for acute stroke patients. In this study skip patterns were used to minimize survey time, mostly completed in 10–15 min. Fatigue may bias patients towards selecting earlier-appearing items. The observed response patterns mitigate this concern. Many early-appearing and mid-list items were not selected by majority of patients, and all questions with lists had at least some patients selecting the last-appearing items. Internal consistency of responses also mitigates the concern: e.g., priority reasons for EMS use showed correlation with recommendations to increase EMS use. Another study limitation, single-center study had a positive effect of enabling maximum diligence in severity data extraction from EMRs, enabling robust severity adjustments. Multi-center studies have 25–30% missing stroke severity data [13].

## Conclusion

Survey findings indicate a widespread absence of knowledge of stroke symptoms and of the importance of EMS use for quick treatment and good outcomes. Knowledge of these factors appears to be unchanged since the 1990s, calling for systematic provider-driven and public health efforts to educate high-risk patient populations (with chronic comorbidities and lifestyle risk factors) as well as the general population to increase EMS use and reduce the prevalence of post-stroke disability. It may be useful for medical, nursing and physician assistant professional organizations to review care guidelines and include stroke education as a standard of care for patients with stroke risk factors including otherwise healthy patients with lifestyle factors such as smoking and alcohol/drug use. The finding of vague/incorrect phone guidance by some physician office staff when contacted by patients at stroke onset indicates the need for medical professional organizations to institute standardized training of physician office staff in phone guidance protocols for stroke and other emergent medical symptoms.

## Supplementary information

**Additional file 1.** Acute ischemic stroke patient survey instrument.

## Data Availability

The data were collected by the authors for the current study and are not made publicly available. Analysis on other hypotheses and unused survey data are continuing, but the data used for the study can be made available by the corresponding author on reasonable request.

## Abbreviations

AIS: acute ischemic stroke
EMS: emergency medical services
GWTG: Get With The Guidelines
NIHSS: National Institutes of Health Stroke Scale
