# Supplementary material for: Why acute ischemic stroke patients in the United States use or do not use emergency medical services transport? Findings of an inpatient survey
Source: BMC Health Serv Res. 2019 Dec 3;19:929. doi: 10.1186/s12913-019-4741-6 (PMC6892139; doi:10.1186/s12913-019-4741-6)
Supplement: Supplementary file 1 — Additional file 1. Acute ischemic stroke patient survey instrument. [file 12913_2019_4741_MOESM1_ESM.pdf]

Appendix 1. Acute ischemic stroke patient survey instrument

(Preferred survey mode – self-administered privately on a computer tablet, with bystander nurse-interviewer available to clarify items when asked.) Stars indicate required items for all surveys.

I. To be completed by Surveyor:
***Surveyor name: ________** ***Verbally verified surveyed patient name with medical record:** Y/N

***Medical Record number: _______** ***Patient consent obtained:** Y/N ***Survey date: ______** **Time: ______**

Date/Time of admission: __/__/__ ___AM/PM ***Survey Respondent:** __ Patient __ Next of Kin (select 1)

***Interviewee if Next of Kin:** ___ relationship to patient ***Age of respondent:** __years

***Reason for next of kin interview:** ___NIHSS≥15 (verify from chart) __ Aphasia present (verify from chart); Patient too tired, not feeling fit to interview: ___ Yes ___ No ___ Other reason

II: To be completed by patient /respondent

1. What was the FIRST symptom or unusual feeling? (Read all in the list and select only **ONE as you best recall**)

___Sudden numbness **WHERE?** __ Face __ Arm __ Leg
___Sudden weakness **WHERE?** __ Face __ Arm __ Leg
___Unable to move
___Drooping of face/mouth
___Sudden trouble seeing in one or both eyes
___Trouble speaking or slurred speech
___Trouble walking OR lost balance OR lost coordination
___Dizziness
___Severe headache without reason
___Confusion
___Suddenly felt very sleepy
___Slowly lost conciousness or control of body
___Sudden feeling of something very wrong; a strange feeling
___Diagnosed at a medical visit OR relative/other person notices something wrong
___Other Please state symptom: ____
___Don't know/ Don't remember **(Skip to Q7 if selected Unknown)**

2. Time you felt the first symptom? ____Date Time:____ AM/PM ____Unknown

3. Were you awake when symptom started?
___ Yes ___No, I woke up with symptom ___Do not know-Not sure

3a. Follow up if No: How did you feel BEFORE going to bed?
___Normal and conscious ___Not fully normal OR felt drowsy OR buzzed
___Feeling of something wrong ___Don’t know

4. What was your SECOND symptom or unusual feeling?

___Sudden numbness **WHERE?** __ Face __ Arm __ Leg
___Sudden weakness **WHERE?** __ Face __ Arm __ Leg
___Unable to move
___Sudden trouble seeing in one or both eyes
___Drooping of face/mouth
___Trouble speaking OR slurred speech
___Slowly lost consciousness or control of body

___Trouble walking OR lost balance OR lost coordination
___Dizziness
___Sudden felt very sleepy
___Severe headache without reason
___Confusion
___Sudden feeling of something very wrong OR strange bad feeling
___Other Please state symptom:______________________________
___Don't know OR Did not have second symptom

4a. Did 2nd symptom start WITH the 1st symptom? ___ Yes ___No

5. When did the second symptom start? ___ Date ___Time ___ AM/PM

6. DID you think of STROKE when you felt the symptoms?

___Yes, at the first symptom ___Yes, after the second symptom ___ Yes, after symptoms worsened
___ Yes, after a friend/family member told me ___ No, I did not think of stroke

6a) Follow-up: if any "Yes" response to 6: What were your thoughts then? (Choose all that apply)

___It may be stroke because of my health problems below:

6.a.i) Follow up to health conditions:

___Overweight
___Diabetes/sugar problem
___High blood pressure
___High cholesterol
___Heart problems
___Other problem (please state problems) ____________

___It may be stroke because my doctor told me about stroke symptoms and to call 911.
___I thought I can't have stroke because I have no health problems that cause stroke
___I can't have stroke because I thought my problems were under control (sugar, blood pressure, etc)
___I can't have stroke because my doctor had not warned me about stroke, only heart attack
___I felt the symptoms were due to tiredness or stress, or drinks or meds that I had taken
___The symptoms were too mild to be a stroke
___The symptom may be temporary due to: ___ neglecting my diabetes (sugar)
___ neglecting my blood pressure
___Such symptoms happened before, and went away, this may be the same thing
___I (or a family member) had a stroke before; this time the symptom was different
___ Stroke cannot happen to ME, I dismissed the idea
___ I felt scared, and I prayed/tried other thoughts to make it go away

6b) Follow up if "No" to 6: Why did you not think of stroke: (Choose all that apply)

___I did not know about stroke symptoms **(for those who selected this, DON'T ASK Q8)**
___I thought stroke only happens to those who are careless about their health, not me
___Because I do not have sugar or blood pressure problems
___Because my sugar or blood pressure is under control
___Because my doctor had not warned me about stroke, only heart attack
___The nurse at my doctor's office had told me about stroke, but not my doctor
___The symptoms were very mild
___I thought the symptom was due to:
___tiredness or stress; ___ neglecting my diabetes (sugar) or blood pressure;
___having some drinks that day; ___ my pain meds or other pills
___I (or a family member) had stroke before; this time the symptom was different
___Such symptoms happened to me before and went away, this may be the same thing
___Other (please state):____

7. (All patients) What did you do about getting help? (Choose one OR more than one)

___I decided to drive myself to the hospital immediately

___My family member/other person drove me to the hospital immediately
___My family member/other person called the doctor's office
___I/we called our friend or relative who is a nurse/doctor/healthcare professional
___I was alone and I waited till my spouse/family member came home
___I/We decided to call 9-1-1 for an ambulance
___I did not want to call 9-1-1, but my family member/other person felt we should
___I did not want to make a big deal by calling for ambulance
___I don't like to go to a doctor or hospital generally, I did not like to get help from others
___I am afraid of disease and hospitals, and decided to wait and see
___In my family or among my people, we wait and see before going to a doctor or hospital

8. Which stroke warning signs DID you know BEFORE the stroke? (Not asked if they selected first item to 6b)

___ Sudden numbness **WHERE?** __ Face __ Arm __ Leg
___ Sudden weakness **WHERE?** __ Face __ Arm __ Leg
___ Sudden trouble seeing with one or both eyes
___ Drooping of face or mouth
___ Trouble speaking or slurred speech
___ Trouble walking, OR losing balance OR coordination
___ Dizziness
___ Severe headache without reason
___ Confusion
___ Other (please state symptom:) ____
___ Did not know any stroke signs

*__8a)Follow up__ if they selected any symptoms). **How did you know about stroke symptoms?**
___ Family member or friend had a stroke; ___ My doctor/nurse told me about symptoms;
___ Brochure at the doctor's office; ___ Read about it on the internet;
___ Billboard on the side of the road; ___ Church or other gathering; ___ TV commercial;
___ Facebook, twitter, or other social media; ___ Other public place (like the supermarket);
___ Other (please specify): _______________

9. I had a stroke, mini-stroke, or stroke-like event before this stroke ___ Yes ___No

10. I know a friend/ family member/ other person who had a stroke ___ Yes ___No

11. Did you/family member/ other person call 911 for an ambulance?
___ Yes ; __ No - did not call 911 - went to hospital by car
___I did not call 911 - the ambulance was already there

If Yes, ask 11a), 11b), 11c), 11d), 11g) and 11h) (11h is done only if ambulance actually came, see 11d)

***11a) Follow up why you call 911: (Reasons why they called 911 for ambulance)**

Out of the following, please ***Select at least 2 MOST IMPORTANT*** *REASONS WHY YOU CALLED 911. Then RANK (1st to 5th - 1st – Most important reason, 2nd – Next most important and so on. You can change your rank or take off your selection if something else comes up later that were more important.*

Yes	Rank	
__	__	I felt my symptoms could be stroke
__	__	Symptoms were severe and scary
__	__	I live alone, and nobody was there to drive me to the hospital
__	__	A neighbor or family member agreed/insisted to call 911
__	__	I know someone with bad effects due to not calling ambulance
__	__	I knew that arriving at the hospital quickly was important
__	__	I knew others who became disabled or died from stroke
__	__	I felt ambulance was the best way to get care
__	__	My doctor told me to call 911 if I had symptoms

__ __ Nurse told me to call 911 if I had symptoms
__ __ I normally take care of my health and felt I needed care urgently
__ __ I/We had good prior experience with using ambulance
__ __ I have good insurance and knew they could cover the cost
__ __ I have good insurance and had met my deductible
__ __ I was unconscious; others called 911
__ __ No other reason

11b) WHEN did you/other person call 911 for ambulance?

___ As soon as I felt the first symptom
___ After discussing with another person
___After returning home from the place where symptom occurred
___When symptoms became worse
___When a new symptom started
___Next morning because symptom started late at night
___After one or two days because symptom started on weekend

11c) Experience of the 911 call for ambulance: **(Choose all that apply):**

___a) Staff took my phone call promptly; ___b) Staff asked questions about symptoms,
___c) Staff immediately said, they were sending ambulance; ___d) Staff advised about using ambulance
___e) Staff asked about insurance; ___f) Advised me to go to hospital; no ambulance available;
___ g) They would call me back when ambulance is available; ___ h) Advised me to call my doctor

11d) Ambulance arrival time:

___ a) Ambulance did not come; ___b) Ambulance came much sooner than expected;
___c) Came in the expected time given distance; ___d) Somewhat delayed than expected;
___e) Very much delayed; ___f) 911 sent Fire/Police instead of ambulance

11e), 11f) and 13) are follow-ups to those who did not call 911:

11e) Why you did NOT call 911 (Choose all that apply):

___I live alone and was too weak/ unable to call
___Symptoms took a long time to become serious
___ I felt normal; symptoms came and went
___I had no pain, so I did not feel it was urgent or serious
___I live alone and was too weak or unable to call
___My family members were out, and I could not move
___My spouse/other household member insisted we should not call ambulance
___My family members were out, and I could not speak
___I was at work/outside home and waited til I could leave
___ I/family member was treated badly by ambulance staff before
___ I called my doctor and they asked me to come in
___ I called my doctors office and they said to go directly to the ER or hospital
___ There is a long waiting time at the ER anyway, may as well go by car
___ I live out in the country, driving may be quicker than ambulance
___ Insurance may not cover ambulance if my symptom was not serious
___ I was worried about my share of the ambulance cost
___ I have no insurance and will get a big bill
___ My insurance has a high deductible; I had not met my deductible
___ I already owe a lot of money to doctors and hospitals
___ I/my family member already had a large expense or bills before this
___ I did not know that I could have serious problems if treatment is delayed
___ I did not know that reaching by ambulance would get me quick treatment
___ I did not trust the hospital or doctor to do their best to help me
___ Hospitals care about money, and I have no insurance, so I may not get good care
___ The hospital/doctor/nurses may not be nice to me
___ The hospital/doctor/nurses may harm me
___ The hospital/doctor/nurses may embarrass me

___ Because I had neglected my blood pressure, sugar or weight problem, ER staff may judge me badly
___ Because I smoke, I did not want to face the hospital staff
___ Because I drink, I did not want to face the hospital staff
___ Because I use drugs, I did not want to face the hospital staff
___ My family/friends may blame me for the stroke, I did not want to face that
___ The ambulance siren and lights will disturb neighbors, I did not want them to know my problems
___ I was not properly dressed to meet ambulance staff

11f) Follow up to selections of 11e). *Here are the reasons why you did not call 911.* *(if they select >5 ask them to select and rank, if not ask them to rank selections only)*

Please select at least 2 most important and RANK 1st to 5th the most important 5 items: 1st – Most important reason why you did not to call, 2nd – Next most important and so on.
(Survey brings up their selected items only).

___Symptoms took a long time to become serious
___I had no pain, so I did not feel it was urgent or serious
___I felt normal; symptoms came and went
___I live alone and was too weak to call
___My family members were out
___My spouse/other household member insisted we should not call ambulance
___I was at work/somewhere else and waited til I could leave
___I called my doctor and they asked me to come in
___I called my doctor and they said to go directly to the hospital or ER
___There is a long waiting time at the ER anyway, so might as well go by car
___I live out in the country, so driving may be quicker than ambulance
___I/family member was treated badly by ambulance staff in the past
___Insurance may not cover ambulance if my symptom was not serious
___I was worried about my share of the ambulance cost
___I have no insurance and will get a big bill
___My insurance has a high deductible; I had not met my deductible
___I already owe a lot of money to doctors and hospitals
___I/my family member already had a large expense or bills before this
___I did not know that I could have serious problems if not treated quickly
___I did not know that reaching by ambulance would get me quick treatment
___I did not trust the hospital or doctor to do their best to help me
___Hospitals care about money, and I have no insurance, so I may not get good care
___The hospital/doctor/nurse may not be nice, or may harm or embarrass me
___Because I have neglected my sugar or blood pressure, the staff may judge me harshly
___Because I smoke/drink/use drugs, I did not want to face the hospital staff
___My family/friends may blame me for the stroke and I did not want to face that
___The ambulance siren and lights will disturb neighbors, and I don't want them to know my business
___I was not properly dressed to meet ambulance staff
___No other reason

11g) Your ER experience on arrival:

11.g.i. How long did you wait in the ER before they took you for treatment?
___hours __ minutes __Don’t know
*__11.g.i.a.__ Did a doctor or nurse check or treat you?
___Yes __No, I left from the first hospital and then came to Richland Hospital

11.g.ii. Did ER staff give you priority for treatment __Yes __No
***11g.ii. a) Follow-up if No: Why? (Choose all that apply) – Because:**
__ I did not come by ambulance
__ I did not tell the important symptoms on arrival.
__ Many waiting patients were in the ER, crowded ER
__ Other patients had more serious-looking symptoms or in pain

___I often used this ER for minor problems.
__ I had no insurance
__ I have Medicaid
__ Because of my race
__ I am poor and they may have judged that I cannot pay

11h) Follow-up if the ambulance came (see 11d): Experience with ambulance
___ Ambulance staff checked me and took me to the Palmetto Richland hospital
___Ambulance first took me to another hospital. Which hospital: ________________.
___Ambulance staff checked me, gave treatment, and told me to contact my regular doctor
___I refused to get checked by ambulance staff and came here by car
___I refused to get checked even the second time the ambulance came and I sent it back
___Ambulance staff was rude, and I decided not to ride the ambulance

12. Previous experience with 911 for ambulance:
Had you or (family) called 911 for ambulance before? ___Yes ___ No;
If yes,
12a) How many times did you/family member/ other person call for ambulance in the last 3 years?
___ Yes ___ No
12b) Follow-up Did you/ family/ other person actually use the ambulance? ___ Yes ___ No
12c) Follow-up Did you any time call for ambulance, but they did not send one? ____Yes __No
12d) Follow-up Did insurance FULLY cover the ambulance cost?
__Yes, every time __Covered fully at times ___I always paid some out of pocket
12d.i) Follow-up If you paid out-of-pocket, how much did you pay or owe the last time you used ambulance? ________________

13. I would recommend my relatives and friends to call the ambulance if they have serious symptoms.
__Strongly agree __Agree __Not sure __Disagree __Strongly disagree

14. I would recommend that anyone with stroke warning signs should call for an ambulance.
__Strongly agree __Agree __Not sure __Disagree __Strongly disagree

15. Based on your own experience, please give 3 SUGGESTIONS to INCREASE ambulance use for stroke?
___ No idea/cannot say
___ More TV commercials about symptoms, diseases that cause stroke, and to call 911 immediately
___ Brochures in doctor's offices about stroke, and to call 911 for symptoms even if living near a hospital
___ Doctors should educate patients about the stroke warning signs and to call 911 immediately.
___ Ambulance staff should be trained to treat all patients with respect and a good attitude.
___ Insurance should fully cover ambulance for stroke symptoms.
___ Other ____

Who actually completed this survey?
___ Patient only
___ Relative only
___ Both
___ Survey not complete due to reason out of control

Appendix 2. Comparison of survey respondents and non-respondents

		Respondents (n = 108) No (%)	AIS patients not surveyed (n = 1071) No (%)
**Demographics and stroke severity**			
Sex*	Male	47 (48.5)	560 (52.3)
	Female	50 (51.5)	511 (47.7)
	Missing†	11	0
Age, mean (SD)*		63.5±15.4	67.6±13.9
Race*	White/Asian/Other	54 (55.7)	536 (49.2)
	Black/African American	43 (44.3)	533 (48.9)
	Missing†	11	2
Severity based on initial NIHSS*	Mild (NIHSS 0-5)	57 (59.4)	625 (60.4)
	Moderate (NIHSS 6-15)	27 (28.1)	250 (24.2)
	Severe (NIHSS ≥16)	12 (12.5)	160 (15.5)
	Missing NIHSS†	12	36
**Medical record-documented arrival mode and alteplase receipt**			
Arrival mode*	EMS vehicle from home/scene	48 (49.5)	557 (52.3)
	Private transport/taxi/other	21 (21.6)	212 (19.9)
	Transfer from other hospital	28 (28.9)**	297 (27.9)
	Missing†	11	5
Alteplase initiated at this hospital*	Yes	14 (14.4)	104 (9.7)
	No	83 (85.6)	967 (90.3)
	Missing†	11	0

AIS, acute ischemic stroke; EMS, Emergency Medical Services; GWTG; Get-With-the-Guidelines; NIHSS, National Institutes of Health Stroke Scale.

* $P < 0.05$ between surveyed and not surveyed AIS patients.

** 27 of them reported using EMS ot reach the first hospital. This information is not captured in GWTG-stroke data.

† Of these, 11 were first admitted to another hospital and transferred to the study hospital, for whom data on sex, race, initial stroke severity, alteplase administration and documented arrival mode was missing in the GWTG registry-sourced data. For all 97 surveyed patients linkable to the GWTG data, their self-reported arrival mode in the survey matched the documented arrival mode in the registry database.

Appendix 3. Responses to survey questions

	Classified by EMS Use		All surveyed Patients n = 108 No (%)
	Yes (n = 75) No (%)	No (n = 33) No (%)	
**Symptoms**			
Stroke symptoms experienced ≥ 1 typical symptom<br>Multiple typical symptoms<br>No typical stroke symptom	33 (44.0)<br>29 (38.7)<br>13 (17.3)	16 (48.5)<br>12 (36.4)<br>5 (15.2)	49 (45.4)<br>41 (38.0)<br>18 (16.7)
Sudden symptom onset	48 (64.0)	24 (72.7)	72 (66.7)
Thought of stroke and perceived symptom as relevant and indicating possible stroke (not dismissing the symptom)*	67 (89.3)	7 (21.2)	74 (68.5)
Awake at stroke onset	60 (80.0)	21 (63.6)	81 (75.0)
Multiplicity of symptoms: multiple symptoms at onset	74 (98.7)	31(93.9)	105 (97.2)
**Did Patient First Think About Stroke?**			
Thought symptom could be stroke: On their own	36 (48.0)	13 (39.4)	49 (45.4)
On being prompted by family member/friend	4 (5.3)	2 (6.1)	6 (5.6)
Did not think of stroke at first	33 (44.0)	17 (51.5)	50 (46.3)
**Knowledge of Stroke Symptoms Before the Stroke**			
Knew 1-3 typical symptoms before the stroke<br>Knew 4-6 typical symptoms before the stroke<br>Knew no symptoms before the stroke	23 (30.7)<br>25 (33.3)<br>27 (36.0)	12 (36.4)<br>12 (36.4)<br>9 (27.3)	35 (32.4)<br>37 (34.3)<br>36 (33.3)
Knew atypical symptoms before the stroke<br>No atypical symptoms before the stroke	44 (58.7)<br>31 (41.3)	17 (51.5)<br>16 (48.5)	61 (56.5)<br>47 (43.5)
**Familiarity with the stroke experience**			
History of stroke/mini-stroke or experience of family member /friend with stroke)*	60 (80.0)	19 (57.6)	79 (73.2)
**Knowledge of Importance of Quick Treatment/Ambulance in Stroke Outcomes**			
Knew the importance of quick treatment/ambulance arrival for good outcome*	27 (36.0)	0	27 (25.0)
**Influence of Social Networks**			
Family member/other person was present at time of stroke	74 (98.7)	33(100.0)	107 (99.1)
Family member/bystander discouraged patient from calling 911*	2 (2.7)	8 (24.2)	10 (9.3)
Family member/bystander supported patient thoughts to call 911*	33 (44.0)	0 (0)	33 (30.6)
**Experienced Symptom Matched Known Symptom**			
Experienced stroke symptom matched knowledge of that symptom	37 (49.3)	16 (48.5)	53 (49.1)
**Financial Barriers/Concerns About Ambulance Use**			
Reported financial concerns about ambulance use /Concern about cost of ambulance use	11 (14.7)	10 (30.3)	21 (19.4)
**Expecting ED care Delay/Prior Experience of ED Waiting Time**			
Prior experience of or expectation of long ER waiting time*	0	2 (6.1)	2 (1.9)
**Live out in the country**			
Live out in the country, better to drive personally to reach quickly*	1 (1.3)	9 (27.3)	10 (9.3)
**Habits of Personal Health Action-Taking**			
Tend to be proactive about personal health/takes care of health	3 (4.0)	0	3 (2.8)
**Concerns about ED Medical Staff's Negative Affective Response**			
Concerned about potential ER medical staff's negative affective response due to personal health habits or other reasons	0	0	0
**Role of Personal Physician or Their Staff**			
Patient reported being educated about stroke symptoms by their	27 (36.0)	10 (30.3)	37 (34.3)

doctor or nurse			
Physician's office directed the patient to actions other than calling 911 when symptoms occurred*	0	6 (18.2)	6 (5.6)
**Source of stroke knowledge**			
Physician/Nurse/ Personal stroke experience	36 (48.0)	19 (57.6)	55 (50.9)
Public sources (internet, billboards, etc.)	23 (30.7)	7 (21.2)	30 (27.8)
No stroke knowledge	16 (21.3)	7 (21.2)	23 (21.3)
**Previous Experience with Ambulance**			
Prior experience of self/family with calling 911 for ambulance*	51 (68.0)	12 (36.4)	63 (58.3)
Had a good experience	1 (1.3)	0	1 (0.9)
Had a bad experience	2 (2.7)	1 (3.0)	3 (2.8)

EMS, emergency medical services; ER, emergency room.

* $P < 0.05$ between the two groups.

Appendix 4. Multiple regression analysis results showing adjusted associations of each factor with EMS use (n = 97)*

	Adjusted Odds Ratio (95%CI)
**Stroke Severity**<br>Initial NIHSS score (continuous variable)	1.2 (1.0, 1.3)†
**Symptoms**<br>Thought of stroke and perceived it as relevant for self having stroke: Yes (vs. No)	41.4 (9.1, 187.8)†
**Experience/Personal History of Stroke**<br>Prior personal or family/friend experience with stroke: Yes (vs. No)	5.8 (1.1, 30.5)†
**Influence of Social Networks**<br>Family member present discouraged the patient from call 911: Yes (vs. No)	0.02 (0.002, 0.3)†

AIS, acute ischemic stroke; ER, emergency room; NIHSS, National Institutes of Health Stroke Scale; OR, odds ratio.

* Adjusted for age, sex and race. N=97 because 11 patients had missing race data. Final model retaining only significant variables is presented. Certain survey items showing differences in bivariate analyses did not attain significance or resulted in indeterminate values (OR.999) due to zero or very low frequencies in the comparison category. These included: Knew the importance of quick treatment/ambulance arrival, Family member/others available at time of stroke, Positive reinforcement to call 911 by person around at the time, Experience or expectation of having to wait in ER, Live out in the country, Tend to be proactive about personal health, Directed by physician's office to actions other than calling 911. Insurance status (Yes/No) was not included as it was not significant in the adjusted model.

† All $P < 0.05$ between the decision to call 911 for an ambulance (Yes/No).

Appendix 5. Patients' EMS Experiences: Current episode and previous experiences before the stroke

	No. (% of total 108 respondents)
**Experience of calling 911 in the current episode**	
EMS staff checked patient and took him/her to the study hospital directly	48 (64.0)
EMS staff transported patient to another hospital first	16 (21.0)
**Prior experience of calling 911**	
No prior experience of calling 911	40 (37.0)
Had prior experience with 911	61 (56.5)
**Previous EMS use experiences**	
Ambulance did not come	2 (3.2)
Cost was covered fully every time	39 (63.9)
Cost was not covered fully at times	7 (11.5)
I always paid some cost out of pocket	14 (23.0)

EMS, emergency medical services.

Appendix 6. Patient recommendations to increase EMS vehicle use by stroke patients*

	No. (% of total 108 respondents)
Insurance should fully cover ambulance for stroke symptoms	50 (46.3)
Doctors should educate patients about the stroke warning signs and to call 911 immediately	46 (42.6)
Brochures in doctor's offices about stroke, and to call 911 when faced with symptoms even if living near a hospital	42 (38.9)
TV commercials about stroke symptoms and diseases that cause stroke, and to call 911 immediately	39 (36.1)
Ambulance staff should be trained to treat all patients with respect and a good attitude	38 (35.2)
Other	14 (13.0)
No response	25 (23.2)

EMS, emergency medical services. *Includes multiple responses, numbers add up to >108.

Appendix 7. Reasons for not completing the survey among survey-eligible AIS admissions

		No.	Percent of total	
Completed survey		108	9.8%	
Patient not contacted (weekends and university breaks)		347	31.5%	
Attempted, but patient found discharged at:	1st attempt*	374	34%	46.9%
	Subsequent attempts	141	12.9%	
Patient not available and family not available for proxy interview		62	5.6%	
Patient refused (including too long consent form)		20	1.8%	
Other reason (incl. ICU-admitted, expired or assigned hospice, aphasic, not English speaking, other)		48	4.4%	
**TOTAL**		1100**	100%	

*45.2% of patients had a length of stay of 4 days or less. With intensive clinical care procedures or patients recovering from the acute aftermath of a brain attack typically taking up to 3 days (patient is not cleared by nursing staff for survey under these conditions), our finding of 34% of patients found to be already discharged by the time of first attempt to contact is consistent with the percent of patients discharged within 4 days or less.
** Total eligible is less than 1179 because the survey period, July 5, 2016 to March 12, 2018 was different from the patient outcomes study period, April 1, 2016 to October 31, 2017.
